# Evaluating a Pivot-Based Approach for Bilingual Lexicon Extraction

**DOI:** 10.1155/2015/434153

**Published:** 2015-04-23

**Authors:** Jae-Hoon Kim, Hong-Seok Kwon, Hyeong-Won Seo

**Affiliations:** Department of Computer Engineering, Korea Maritime and Ocean University, 727 Taejong-ro, Yeongdo-gu, Busan 606-791, Republic of Korea

## Abstract

A pivot-based approach for bilingual lexicon extraction is based on the similarity of context vectors represented by words in a pivot language like English. In this paper, in order to show validity and usability of the pivot-based approach, we evaluate the approach in company with two different methods for estimating context vectors: one estimates them from two parallel corpora based on word association between source words (resp., target words) and pivot words and the other estimates them from two parallel corpora based on word alignment tools for statistical machine translation. Empirical results on two language pairs (e.g., Korean-Spanish and Korean-French) have shown that the pivot-based approach is very promising for resource-poor languages and this approach observes its validity and usability. Furthermore, for words with low frequency, our method is also well performed.

## 1. Introduction

Bilingual lexica are very important resources for bilingual works like machine translation, cross-language information retrieval, foreign language learning, terminology-related studies, and so on. Therefore, many researchers [1–4] have attracted bilingual lexicon extraction (BLE) and have proposed several approaches under various circumstances. The approaches can be broadly classified into three categories based on the different usage of language resources like corpora and dictionaries.

The first category is the simplest approach using a machine readable dictionary (MRD) [5]. This approach directly extracts them from MRDs or Web-based dictionaries like Wikitionary (http://www.wiktionary.org/) and Wikipedia (http://www.wikipedia.org/). It is very simple, but there are no MRDs in some language pairs (in particular, resource-poor language pairs like Korean-Bengali). Besides, many MRDs are not well formed like XML, although MDRs might be, and it is difficult to extract them from MDRs.

The second category is an approach using parallel corpora [6]. This approach extracts translation equivalents from parallel corpora based on word alignment models, which are well established [7, 8]. This approach is relatively high in the quality of extracted bilingual lexica if the size of parallel corpora is large enough [6]. Also this approach is very practical and easily applicable since there are several word alignment tools publicly available (http://web.eecs.umich.edu/~mihalcea/wa/). However, the effectiveness of the methods seems to be highly dependent on the availability of sizeable and high-quality parallel corpora. Also most parallel corpora are only provided for well-known language pairs like English and Spanish and the existing parallel corpora do not cover most domains [9].

The third category is an approach using comparable corpora and seed dictionaries [2, 3]. This approach extracts translation equivalents by comparing source context vectors and target context vectors which are made from comparable corpora. In fact dimensions of the two vectors are different from each other and source context vectors have to be translated in target languages using seed dictionaries in order that the dimensions agree. Recently this approach has been most widely used. Comparable corpora cause the accuracy of BLE to be lower than the ones obtained from parallel corpora of similar sizes [2] and the applicability of this approach depends on the size of the initial seed dictionary which is needed for translating target context vectors [2, 10]. The size is important to achieve good applicability because the larger the size is, the higher the applicability is. Hence, some researchers [11] have studied extending the initial seed dictionary automatically. Since the quality of the extracted bilingual lexica is relatively sensitive to the type of corpora used in the extraction process, one would be naturally skeptical with the ideas of building a bilingual lexicon automatically using comparable corpora [9].

One common thing among those studies is that corpora like comparable and/or parallel corpora are essentials for building bilingual lexica. Unfortunately, for some language pairs like Korean and Bengali, it is not easy to get such corpora in the public domain. What was worse, it is difficult to build such corpora because it is very expensive and tedious to build for new language pairs parallel and/or comparable corpora needed for extracting the bilingual lexica. Just like in the corpora, there is the same difficulty for the initial seed dictionaries of such language pairs. In order to alleviate these problems, some researchers proposed pivot-based approach [12–17]. There are three different phases in pivot-based approaches. The first is to combine two bilingual dictionaries sharing with one common language as a pivot language, for example, Japanese-English and English-Chinese dictionaries (to build a Japanese-Chinese dictionary) [12, 17]. The second is to merge two phrase tables sharing with one common language as a pivot language [15]. The two phrase tables are extracted from two parallel corpora on the basis of phrase based statistical machine translation (SMT). The third is to build bilingual lexica based on the similarity of context vectors in a pivot language [13, 14]. The context vectors (called source and target context vectors) in this approach are represented by words in a pivot language and are produced using two parallel corpora sharing the pivot language, that is, source-pivot and pivot-target parallel corpora. We call this approach a pivot-based standard approach (PBSA). This paper is related to this approach, which will be described further in detail.

In this paper, we will evaluate the PBSA in company with two different methods for estimating context vectors represented by words in a pivot language (called pivot-based context vectors). The purpose of this paper is twofold: one is to show the validity of pivot-based context vectors, which are produced by the two estimation methods. The other is to show the usability of the PBSA in between resource-poor language pairs to extract bilingual lexica.

The paper is organized as follows: Section 2 describes the related works of the standard approach and the PBSA, from which our works are derived. Then, Section 3 presents two estimation methods of context vectors and Section 4 describes some experiments and discussions on Korean-French and Korean-Spanish lexicon extraction. Finally, Section 5 draws conclusions and discusses some perspectives on our future works.

## 2. Related Works

There are many works focused on extracting bilingual lexica from comparable corpora [3, 18–20]. Most of them are based on the context-based approach, so called the standard approach, proposed by Rapp [10]. In this section, first of all, we describe the standard approach (SA) in short and then the pivot-based standard approach (PBSA) proposed by Kim et al. [13, 14].

### 2.1. Standard Approach

The basic assumption of the standard approach is distributional hypothesis [21], which states that words with a similar meaning are likely to appear in similar context across languages. Therefore, a word can be represented as a context vector of similar words. Under BLE from comparable corpora, there are two context vectors, source and target context vectors for source and target words, respectively. Each element in the context vectors represents its association with a word which occurs within a window of words. The two vectors, however, are incomparable because the dimensions of them are totally different from each other. That is, one is represented by words in a source language and the other in a target language. In order to enable the comparison of source and target context vectors, words in the source context vectors (or target context vectors) are translated into the target language (or source language) using a seed bilingual dictionary between the source language and the target language. A vector similarity like cosine similarity and Jarcard coefficient is used and target words with the highest vector similarity are treated as translation candidates. In summary, the standard approach can be carried out by applying the following four steps:

1. context characterization: to build source and target context vectors for each word of source and target languages, respectively. All words in the context vectors can be weighted with an association measure like *χ* ^2^ score,
2. context vector translation: to translate words in a source context vector in a target language using a seed dictionary,
3. similarity calculation: to compute similarity between the translated context vector and all target context vectors through vector distance measures such as cosine similarity,
4. candidate translation selection: to rank translation candidates according to the similarity score.

The advantage of the standard approach is that it is a fast and affordable way to construct bilingual lexica in resource-poor language pairs and new domains [22]. However, it also presupposes the availability of a seed bilingual dictionary to translate context vectors, which is not the case for many language pairs or domains. Hence, some researchers had extended the seed dictionary automatically. Comparable corpora are also essentials. Unfortunately, for some language pairs like Korean and Bengali, it is not easy to get such corpora in the public domain.

### 2.2. Pivot-Based Standard Approach

**Figure 1:**
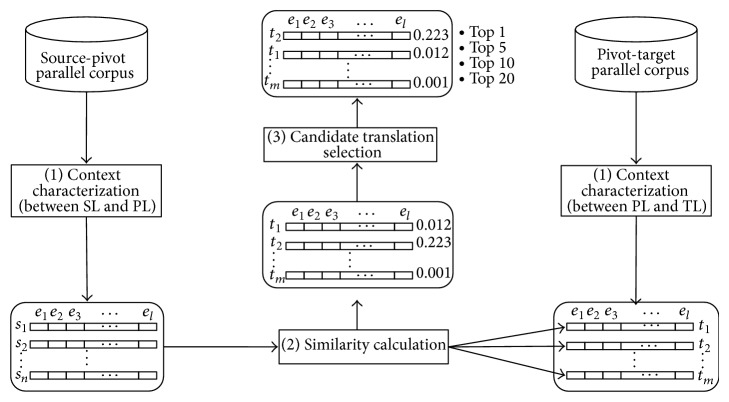
Overall structure of the PBSA for BLE (adapted from the paper [13]).

As mentioned in the previous section, there are many advantages in the standard approach, but the standard approach still has some problems on resource-poor language pairs like Korean and Bengali. To overcome these problems, the PBSA was proposed by Kim et al. [13, 14], with adapting from the standard approach.  Figure 1 is the overall structure of the PBSA, where *s*_*i*_, *t*_*i*_, and *e*_*i*_ are the *i*th word in a source language, a target language, and a pivot language, respectively, and *n*, *m*, and *l* are also the number of words in a source language, a target language, and a pivot language, respectively. As shown in Figure 1, unlike the standard approach mentioned before, there are three steps: (1)* context characterization*, (2)* similarity calculation*, and (3)* candidate translation selection*. That is, there is no step of context vector translation using a seed dictionary. The second step of similarity calculation and the third step of candidate translation selection are the same as those of the standard approach, but the first step of context characterization is totally different from that of the standard approach. In the PBSA, context vectors are made up of words in a pivot language as a bridge language, which should be very popular like English. We call the words “pivot words” hereafter. That is, both of source and target context vectors are represented by pivot words instead of their own words. As a result, they have the same dimensions and can be compared with each other to get similarity between them. Now the remaining problem is how to get the context vectors. To find out probable pivot words for a source word (resp., target word), in this paper, we use two parallel corpora, a source-pivot parallel corpus (SL-PL parallel corpus) and a pivot-target parallel corpus (PL-TL parallel corpus). If the parallel corpora could be publicly available, pivot-based context vectors should be easily estimated through word alignment [9, 23, 24]. In this paper, we evaluate two estimation methods of the context vectors, which are described in the next section.

Table 1 shows the difference between the standard approach and the PBSA. The PBSA has much strength in terms of a seed dictionary, context vector translation, and computational efficiency, but some weaknesses as to domain adaptation and corpus preparation. The PBSA does not use any linguistic resources such as seed dictionaries except parallel corpora sharing a pivot language. We can obtain more accurate alignment information by using parallel corpora instead of comparable corpora.

## 3. Estimation Methods of Context Vectors

In the PBSA using two parallel corpora, SL-PL and PL-TL, estimating a context vector for a given source word (resp., target word) is the same as aligning source words (resp., target word) into pivot words in the SMT. If a bilingual dictionary between a source language (resp., target language) and a pivot language is publicly available, it could be used for estimating the context vector. Generally it is not simple to get such a bilingual dictionary and also to build it from an MRD because the MRD is not well formatted in a markup language like XML. Accordingly, most of works on word alignments are based on cooccurrence [25, 26]. We evaluate two methods for estimating context vectors based on word cooccurrence. One is based on a word alignment tool, which is publicly available and can produce translation probabilities. The other is based on word association between source words (resp., target words) and pivot words in an SL-PL parallel corpus (resp., PL-TL parallel corpus) through a contingency table.

### 3.1. Word Alignment Tools

One of the word alignment tools like Moses (http://www.statmt.org/moses/) and Anymalign (https://sites.google.com/site/adrienlardilleux/) can be used for estimating a source context vector (resp., target context vector) for each word *s*_*i*_ (resp., *t*_*i*_) in a source language (resp., target language) using an SL- PL (resp., PL-TL) parallel corpus. In this paper, we use Anymalign [23] as a word alignment tool. For a parallel corpus, Anymalign can produce bidirectional translation probabilities, that is, *Pr*⁡⁡(*e*_*k*_∣*s*_*i*_) and *Pr*⁡⁡(*s*_*i*_∣*e*_*k*_), where *s*_*i*_ and *e*_*k*_ are the *i*th word and the *k*th word in the source language and the pivot language, respectively. The size of context vectors estimated in such a way is very large because of some noises like improper translations. In order to reduce the noises and improve the reliability of the context vectors, consider Table 2 partial examples of translation probabilities between Korean words as source words and English words as pivot words.

In Table 2, the first row is a good example, but the rest of rows are not. In case of the second row, it is much less possible that *s*_*i*_ and *e*_*k*_ may be translation equivalents because the difference between two probabilities is too big. In case of the third row, it is also improper translation because the two probabilities are too low. In summary, an association between *s*_*i*_ and *e*_*k*_, *a*(*s*_*i*_, *e*_*k*_) can be estimated as follows:

(1) $$\begin{array}{c} a\left(\;s_{i},e_{k}\;\right)=\left\{\begin{array}{cc} \frac{\mathrm{Pr}\left(e_{k}\;|\;s_{i}\right)+\mathrm{Pr}\left(s_{i}\;|\;e_{k}\right)}{2} & \text{if}\;\;\mathrm{Pr}\left(e_{k}\;|\;s_{i}\right)>\theta_{1},\;\;\;\mathrm{Pr}\left(s_{i}\;|\;e_{k}\right)>\theta_{1},\;\;\left|\mathrm{Pr}\left(e_{k}\;|\;s_{i}\right)-\mathrm{Pr}\left(s_{i}\;|\;e_{k}\right)\right|<\theta_{2}\\ 0 & \text{otherwise}, \end{array}\right. \end{array}$$

where *θ*_1_ and *θ*_2_ are constants as thresholds.

### 3.2. Contingency Table

Measures of bilingual word association are used to determine if a word in one language and another word in a second language are dependent on one another or not in a parallel corpus [27, 28]. In our case, the two words can be a source word *s*_*i*_ (resp., a target word *t*_*j*_) and a pivot word *e*_*k*_. To measure the dependence of these two words, we determined their frequency counts using a simple statistical model. We considered the two words to be represented by binary random variables that simply indicate if a word occurred or not in their corresponding pieces. A word pair can fall into one of the four possible categories, and we can represent the associated count data using a two by two contingency table.  Table 3 shows an example of a 2 by 2 contingency table for a Korean word *s*_*i*_ =* kyeong-chal* and an English word *e*_*k*_ =* police*. Measure of association in finding word translations was proposed by Gale and Church [29], who employed the *φ*-coefficient and pointwise mutual information as their measures of association. Since then, several word association measures have been proposed in the literature [26]. In this paper, we make use of *χ* ^2^ score defined as (2) through the 2 by 2 contingency table:

(2) $$\begin{array}{c} \chi^{2}=\frac{{\left[\left(n_{11}\times n_{22}\right)-\left(n_{12}\times n_{21}\right)\right]}^{2}\times n_{++}}{n_{1+}\times n_{2+}\times n_{+1}\times n_{+2}}. \end{array}$$

The *χ* ^2^ value is 24.78 for Table 3 and the critical value is *χ* ^2^ = 3.841 (you can find the *χ* ^2^ distribution table from http://easycalculation.com/statistics/chisquare-table.php). So we reject the null hypothesis that* kyeong-chal* and* police* occur independent of each other. That is, the Korean word* kyeong-chal* and the English* police* can be a good translation candidate.

## 4. Experiments and Evaluation

In order to evaluate the performance of the PBSA with the two estimation methods of context vectors, we perform experiments over two language pairs, Korean-Spanish (KR-ES) and Korean-French (KR-FR). In this section, we first give experimental setups such as parallel corpora and evaluation dictionaries, and then we present the results of experiments conducted on the two language pairs. Finally we analyze some errors and discuss some issues on BLE through these results.

### 4.1. Experimental Environments

#### 4.1.1. Parallel Corpora

We need three parallel corpora, KR-EN, EN-ES, and EN-FR, since we use English (EN) as a pivot language. In the case of the KR-EN parallel corpus, we use the KMU Parallel Corpus [30], which is freely available on the Web (https://sites.google.com/site/nlpatkmu/Resources/Corpora). The parallel corpus was built in 2006 and consists of 433,151 sentence pairs of KR-EN. In the case of the EN-ES and EN-FR parallel corpora, we use the Europarl Parallel Corpus [31], which is also freely available on the Web (http://www.statmt.org/europarl/). Actually we use only subcorpora to keep balance with the KR-EN parallel corpus in size. The two sets of subcorpora of EN-ES and EN-FR consist of 500,000 sentence pairs each that are randomly selected from the original corpus of the Europarl Parallel Corpus. To tell the truth, the KMU Parallel Corpus and the Europarl Parallel Corpus are built from different domains (news article and European Parliament proceedings). The average number of words per sentence in the three parallel corpora is shown in Table 4.

As you can see in Table 1, word distributions are similar except those of KR-EN. The number of Korean words (called Eojeol in Korean) in KR-EN corpus is lower than others. This is caused by the Korean characteristic that Korean words usually have one morpheme or more (average number of morphemes per word = 2.1, but varies with a corpus). Considering Korean morphemes, the number could be similar to that of EN.

#### 4.1.2. Preprocessing

All words are tokenized and tagged in POS (part-of-speech) by the following tools: Hannanum (http://kldp.net/projects/hannanum) [32] for Korean and TreeTagger (http://www.cis.uni-muenchen.de/~schmid/tools/TreeTagger) [28] for English, Spanish, and French. Moreover, all words except content words (nouns, main verbs, adjectives, and adverbs) are removed as stop words and then are converted to lower case.

#### 4.1.3. Evaluation Dictionary

For the evaluation, we use the KMU Evaluation Dictionary for BLE, which is freely available on the Web (https://sites.google.com/site/nlpatkmu/Resources/Lexicons). The dictionary was manually built using the Web dictionary (http://dic.naver.com/) and contains four bilingual lexica, KR-ES, KR-FR, ES-KR, and FR-KR. Each lexicon is unidirectional and contains 200 frequent words (denoted as HIGH) and 200 rare words (denoted as LOW). The frequent words and the rare words in the lexica are randomly sampled from three parallel corpora (mentioned in the previous section) based on the frequency of source words, respectively. Their translation equivalence distribution is described in Table 5. Those are considered as to the degree of ambiguity. Overall, the degree of ambiguity for Korean as a source language is lower than that as a target language without respect to the frequency of HIGH or LOW.

### 4.2. Performance Evaluation

In this section, we discuss the performance of the two estimation methods, a word alignment tool of Anymalign (ANY) and a contingency table for word association of *χ* ^2^ (CHI), which were explained in Section 3.

Each estimation method has model parameters. For ANY, there are two model parameters, *θ*_1_ and  *θ*_2_. *θ*_1_ is fixed to 0.5 for both HIGH and LOW and *θ*_2_ is set to 0.005 for HIGH and 0.003 for LOW through several experiments. On the other hand, for CHI, a model parameter is the critical value of *χ* ^2^ as 3.841 (the *α* level of significance is 0.05 and the degree of freedom is 1 on the the *χ* ^2^ distribution table.) as mentioned in Section 3.2.

To evaluate the quality of translation candidates extracted by systems, we use accuracy (ACC) [33], mean reciprocal rank (MRR) [34], precision (PRE) [35], recall (REC) [35], and rated recall (RRC) [36] as evaluation metrics. ACC and MRR show the performance for at least one correct answer, while PRE, REC, and RRC present the performance for multiple correct answers. The formal definition of each evaluation metrics will be described in subsequent sections.

#### 4.2.1. Performance of Accuracy

Generally, accuracy (ACC) is the fraction of their translation candidates that are correct and we use top-*k* ACC, that is, the proportion of source words which have at least one translation equivalent among top *k* of its induced translation candidates. More formally, top-*k* ACC, ACC_*k*_ is defined as

(3) $$\begin{array}{c} {\text{ACC}}_{k}=\frac{1}{N}\overset{N}{\underset{i=1}{\sum }}\underset{1\leq j\leq k}{\max}a_{ij},\;\text{where}\;\;a_{ij}=\left\{\begin{array}{cc} 1 & \text{if}\;\;t_{ij}\in A_{i}\\ 0 & \text{otherwise}, \end{array}\right. \end{array}$$

where *N* is the number of source words which are evaluated, *A*_*i*_ is a set of translation equivalents for a source word *s*_*i*_, and *t*_*ij*_ for *s*_*i*_ is the *j*th translation candidate, which is induced by a BLE system.

**Figure 2:**
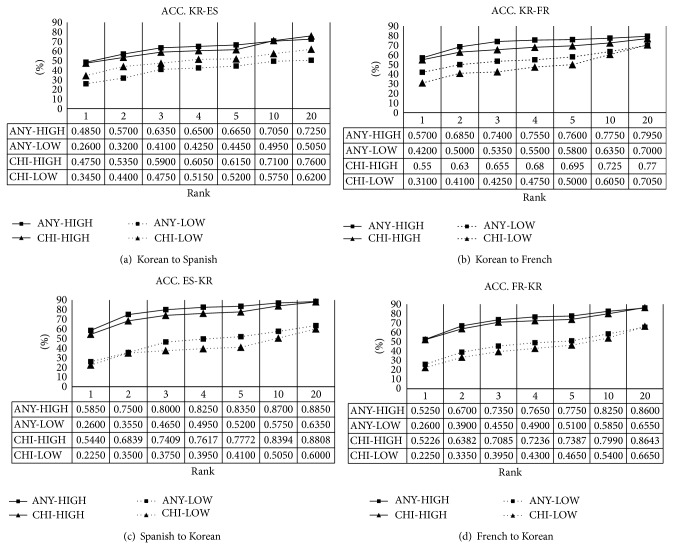
Accuracy of *k* best candidates with different ranks.

Basically, we can show the performance for at least one correct answer through ACC as mentioned before. The results are shown in Figure 2. Overall, ANY a little outperforms CHI in accuracy, but the two methods show similar performance at the top 20. Although it is different in language pair, the minimum and maximum accuracy for HIGH is 48.5% and 58.5% at the top 1, respectively. This means that our system finds the correct translation equivalents for half of high frequent words in a source language at the top 1. As for the ACC in the top 5 for HIGH, the minimum and maximum accuracy is 66.5% and 83.5%, respectively. According to this result, most of the dominating translation equivalents for words are found in the top 1 through 5. Consider the performance in the case of LOW. ACC for LOW is much lower than that of HIGH as you can predict. The maximum at the top 1, however, is 42% in the case of KR-FR. This states that it is not bad even if a word is rare. We believe that the most important thing in BLE is that a system has good performance for rare words as the so-called* hapax legomena*.

#### 4.2.2. Performance of Mean Reciprocal Rank

Mean reciprocal rank (MRR) is derived from question answering [34] and the average of the reciprocal ranks of translation candidates that are correct translations for source words. This definition does not specify what to do if none of the proposed results are correct (use mean reciprocal rank 0), or if there are multiple correct answers in the list (use the best one). Consequently MRR accentuates translation candidates at higher ranks more than others at lower ranks so that the correct translation candidates at higher ranks could be treated as more important. MRR at the top *k*, MRR_*k*_, is formally defined as

(4) $$\begin{array}{c} {\text{MRR}}_{k}=\frac{1}{N}\overset{N}{\underset{i=1}{\sum }}\underset{1\leq j\leq k}{\max}r_{ij},\;\text{where}\;\;r_{ij}=\left\{\begin{array}{cc} \frac{1}{j} & \text{if}\;\;t_{ij}\in A_{i}\\ 0 & \text{otherwise}. \end{array}\right. \end{array}$$

**Figure 3:**
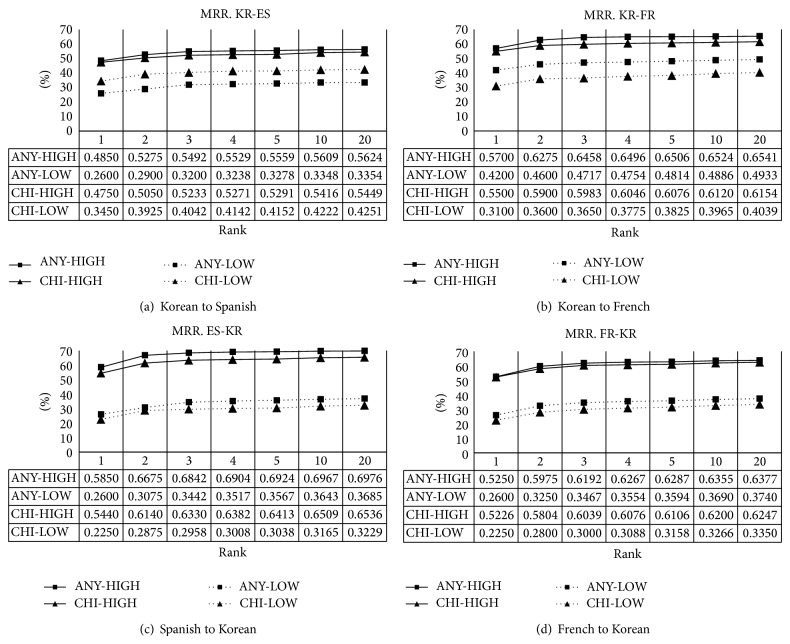
Mean reciprocal rank of *k* best candidates with different ranks.

Basically, like ACC, MRR also reveals the performance for at least one correct answer, but unlike ACC, MRR prefers correct answers at the lower rank (i.e., the top 1) to those at the higher rank. The results are shown in Figure 3. ANY also outperforms CHI in MRR because MRR shows the same characteristics with ACC in general. Consider the slopes of graphs minutely. For all over the graphs, the slopes are steep in the top 5 below and the slopes are gentle in the top 5 above. It indicates that most of correct translations lie below the top 5. We can consider translation candidates at the top 5 above to be quite rare. In this respect, we can tell that the PBSA is very promising.

#### 4.2.3. Performance of Precision

Precision (PRE) (also called positive predictive value) is widely used in information retrieval and is the fraction of extracted translation candidates contained in the evaluation dictionary. Unlike ACC and MRR, PRE can show the performance for multiple correct answers. Formally, PRE at the top *k*, PRE_*k*_, is defined as

(5) $$\begin{array}{c} {\text{PRE}}_{k}=\frac{1}{N}\overset{N}{\underset{i=1}{\sum }}\frac{1}{k}\overset{k}{\underset{j=1}{\sum }}a_{ij},\;\text{where}\;\;a_{ij}=\left\{\begin{array}{cc} 1 & \text{if}\;\;t_{ij}\in A_{i}\\ 0 & \text{otherwise}. \end{array}\right. \end{array}$$

**Figure 4:**
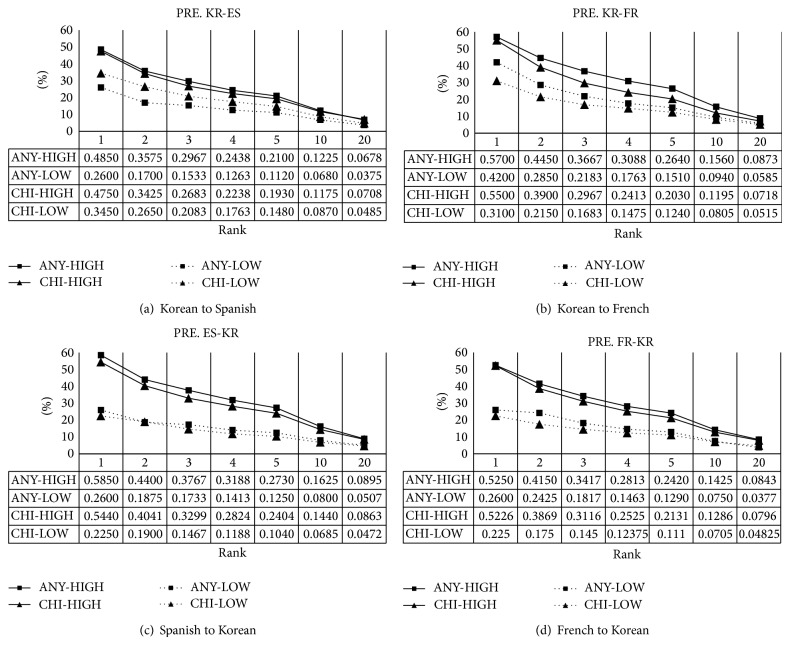
Precision of *k* best candidates with different ranks.

The results are shown in Figure 4. For HIGH, ANY outperforms CHI generally like ACC, but the gap of the performance is not big. Same as in ACC, the minimum and the maximum PREs for HIGH are 48.5% and 58.5% at the top 1, respectively. This means that our system finds the correct translation equivalents for half of high frequent words in a source language in the top 1. The minimum and maximum PREs, however, are 6.78% and 8.95% at the top 20, respectively. This indicates that correct translation equivalents comprise about 7% of extracted translation candidates at the top 20. At a glance, the performance is very low. See the number of ambiguity as the number of multiple answers in Table 4. The average number is in between 5 and 11 and then false alarms in many cases cannot help turning on among the top 20. For LOW, CHI outperforms ANY for KR-ES, but ANY outperforms CHI for the rest. The reason has not been figured out so far, so we need to examine the strange phenomenon in fact.

#### 4.2.4. Performance of Recall

Recall (REC) (also known sensitivity) is also widely used in information retrieval and is defined as the fraction of translation equivalents that the system extracted. Unlike ACC and MRR, REC can show the performance for multiple correct answers like PRE. Formally, REC at the top *k*, REC_*k*_, is defined as

(6) RECk=1N∑i=1N1Ai∑j=1kaij,where  aij=1if  tij∈Ai0otherwise.

**Figure 5:**
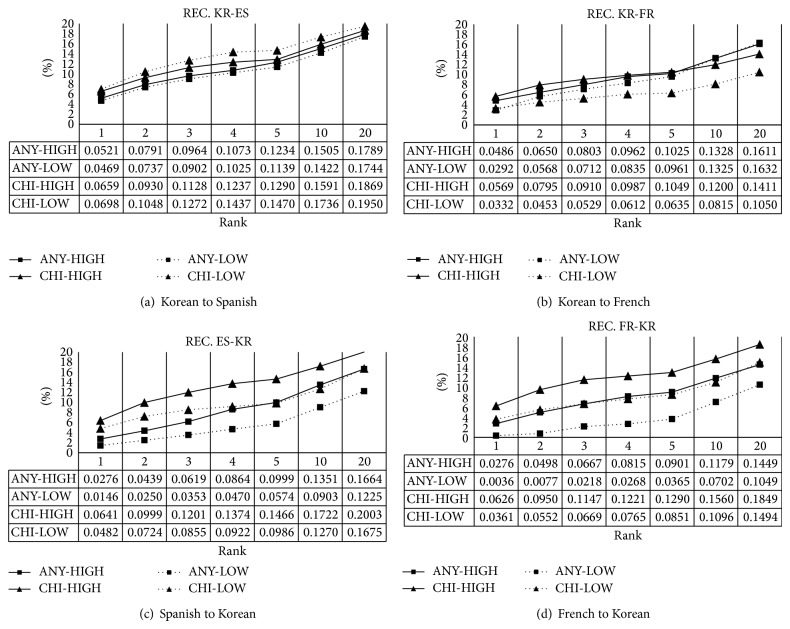
Recall of *k* best candidates with different ranks.

The results are shown in Figure 5. CHI outperforms ANY in REC generally. This is caused by the size of context vectors and the size of context vectors for CHI is generally larger than that for ANY. This phenomenon tells us that CHI is favorable to multiple answers. Also as for REC, slightly different inclination is shown as in Figure 5(a). The performance of LOW is not lower than that of HIGH. Generally words in HIGH are very ambiguous, which means that the words have many translation equivalents in the evaluation dictionary (see Table 5). This has been revealed by Figure 5. On the whole, the performance is quite low from about 4% to 7% at the top 1 and from about 10% to 20% at the top 20. To tell the truth, most of translation equivalents in the evaluation dictionary are not used as the meaning in the corpora. For example, translation equivalents for the Korean word* jeon-ryak* are* strategy*,* tactic*, and* stratagem* in English according to a machine-readable dictionary of Korean-English, but the first one is mainly used in the real text (the frequency of* strategy*,* tactic*, and* stratagem* is 6026, 415, and 48, resp., in the BYU-BNC (http://corpus.byu.edu/bnc/)). Consider the slopes of graphs minutely. For all over the graphs, the gradients are continuously increased although the magnitude is low. It indicates that the higher the rank is the more the correct translation equivalents are found.

#### 4.2.5. Performance of Rated Recall

First of all, consider an evaluation dictionary as a gold standard for evaluating a BLE system. Generally an entry of the dictionary is composed of a pair of source words and one or more translation equivalents (target words). Any translation equivalents do not appear in the training corpus at all and some translation equivalents account for a great part of the training corpus. In the case of the former, there is no way of extracting them under BLE. In the case of the latter, it is important that the high frequent translation equivalents should be found as soon as possible. That is, the frequency of translation equivalents in the training corpus is not reflected at all in performance evaluation so far. In order to supplement such problems, rated recall (RRC) is proposed by Seo et al. [36]. RRC is the proportion of extracted translation equivalents in the training corpus. Formally, RRC at the top *k*, RRC_*k*_, is defined as

(7) RRCk=1N∑i=1N ∑j=1kaijrtij,where  aij=1if  tij∈Ai0otherwise,

where *r*(*t*_*ij*_) is the relative frequency of translation equivalents *t*_*ij*_ for a source word *s*_*i*_ in the training corpus and ∑_*t*∈*A*_*i*__ *r*(*t*) = 1. An example of how to calculate RRC is described in more detail as below.  Table 6 shows Korean translation equivalents for the French word* décision* and their relative frequency. As you can see in Table 6, the Korean translation equivalent* geol-dan-ryuk* cannot be extracted in any case because its relative frequency is zero where it does not appear in the training corpus. We can calculate the RRC_*k*_ for the French word* décision* under Table 6. If* gyuol-jung* and* gyeol-ui* are extracted among the top 5, RRC_5_ is 80% (0.754 + 0.046).

**Figure 6:**
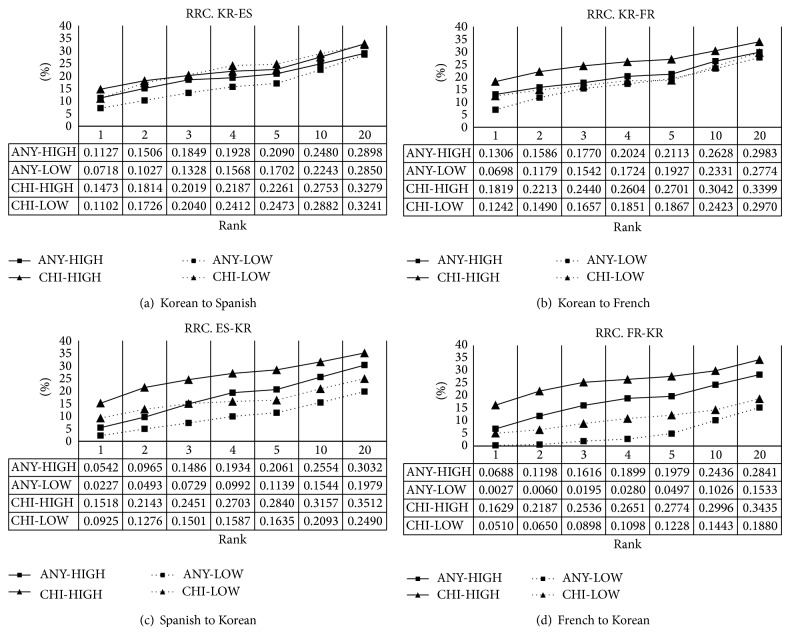
Rated recall of *k* best candidates with different ranks.

The results are shown in Figure 6. As you can see in Figure 6, the performance is much higher compared to the recall shown in Figure 5. As for HIGH, the performance is from approximately 5% to 13% at the top 1 and from approximately 28% to 35% at the top 20. As for LOW, the performance is from approximately 5% to 13% at the top 1 and from approximately 1% to 32% at the top 20. The range is quite big. Overall, CHI outperforms ANY for HIGH and LOW.

### 4.3. Error Analysis

Tables 7 and 8 show Korean translation candidates for the Spanish word* estrategia* (*strategy*) and for the French word* monde* (*world*), respectively. Each table contains correct translation equivalents and some errors on the ranked list. In this section, we consider errors. We present characteristics of Korean word formation by analyzing errors in BLE.

First, many Korean words are derived from Chinese, for example, the Korean word* bu-eob* (its translation equivalents in English are* side   job*,  * by-job*,* sideline*, * side business*,  * subsidiary  work*,* auxiliary occupation*,* minor occupation*, and * sideline occupation* (from http://endic.naver.com/)) derived from a sequence of the Chinese characters 副業. A Chinese character has a meaning because a Chinese character is an ideogram. In the previous example, the meaning of the Chinese character of 副 is* secondary*,* auxiliary*,* subsidiary, and so forth* and that of 業 is* job*,* work*,* occupation, and so forth*. These characteristics make words abundant and consequently the ambiguity of Korean words could be reduced to a certain degree (see Table 5). These characteristics are one of the main reasons of errors in BLE (word-to-word mapping). As you can see in the previous example, two translation equivalents,* sideline *and* by-job*, are comprised of only one word and the rest are comprised of two words. The former can be extracted in BLE, but the latter cannot exactly and only a part of them can be found. In the end, they make errors in bilingual word-to-word lexicon extraction like this work. This is the reason why multi-word expressions should be treated in BLE. In this paper, multi-word expressions as translation equivalents are not included in the evaluation dictionary and then the performance is not influenced by this characteristic.

Second, word spacing is unclear in Korean. Basically a Korean word is separated by a space, so compound words, especially compound nouns, should contain one or more spaces between words. In many case, compound words in Korean real text may be used without any spaces. These characteristics cause errors in BLE. Such errors can be found in Table 7. See the first row for composing* sa-eop* (*business*) and* jeon-ryak* (*strategy*), the fourth row for making up* gi-bon* (*basic*) and* jeon-lyak *(*strategy*), and the fifth for forming* tu-ja* (*investment*) and* jeon-ryak* (*strategy*). All of these contain the basic meaning of* jeon-ryak* (*strategy*), but they are counted as errors. If a tokenizer would separate a compound into basic words, these could be counted as errors. Consequently a good tokenizer (or a good POS tagger) has to be used in order to reduce such errors. Any tokenizer, however, cannot help generating a few errors no matter how good the tokenizer is and they can also affect the performance.

Third, many transliterated words in Korean are used in real text, but not include in the MRD which we used as the evaluation dictionary (gold standard). For example,* weol-deu* (*world*) at the third row of Table 8 is transliterated by substituting the English letters world for Korean letters. This characteristic also causes errors in BLE for Korean.

Fourth, every MRD as a gold standard is incomplete. See the fourth and the fifth row of Table 8. The basic meaning of* se-gye* is the same as that of* jeon-se-gye*. The difference is that the former is usually used as noun and the latter as adjective. The word* gak-guk* involves* every country*, namely,* whole world*. These kinds of words can be treated as correct. To solve this problem, human judgments can be used, but it is too expensive and tedious and it is not effective. At the end, different evaluating method to take into account similar words should be considered.

### 4.4. Discussion on Experiments

The purpose of this work is not to try to find the best results by looking for the best tuning of each variation like model parameters. Here, the main interest is to show the validity of pivot-based context vectors and the usability of the PBSA for resource-poor language pairs. The validity of context vectors in BLE was described by Gaussier et al. [37]. In the same context, pivot-based context vectors are represented by words in a pivot language (as for standard approach, a target language) and can be interpreted as the same geometric view, and our experimental results are its evidence. Also through experimental results, we have observed that the PBSA is useful for resource-poor language pairs. In fact, any linguistic resources between Korean and Spanish can barely be obtained in the public domain, but we were able to build a bilingual lexicon between two languages. It is important to say that the PBSA is conceptually naive. The process of looking for translation equivalents does not take into account any linguistic resources like seed dictionaries; it is just based on publicly available parallel corpora that are sharing a resource-rich language as a pivot language. But, this approach in performance is worth comparing with other approaches in BLE.

Consider two estimation methods, ANY and CHI.  Table 9 demonstrates comparison of the performance in two estimation methods, ANY and CHI. In the table, the cell marked ANY shows that ANY outperforms CHI, on the contrary, the cell marked CHI shows that CHI outperforms. As you can see in the table, on the whole, although there are some exceptions, ANY outperforms CHI in accuracy like ACC, MRR, and PRE, while CHI outperforms ANY in recall like REC and RRC. Unusually for KR-ES of LOW, CHI is always superior in performance. We are seriously thinking about the reasons. We believe that the distinction between ANY and CHI results from the size of context vectors as mentioned in Section 4.2.4. Context vectors for ANY are short in size and noise elements in the vectors are small in number compared with CHI.

In BLE, effective evaluation methods should be developed because a MRD as a gold standard is not sufficient as mentioned in Section 4.3. In general the MRD which is publicly available does not reflect real text of the day, but also we cannot say that the dictionary is complete.

Almost always, systems for Korean as a source language outperform those for Spanish and French. We believe that this is due to the ambiguity of translation equivalents. As you can see in Table 4, the ambiguity for Korean as a source language is lower than ambiguity of others.

## 5. Conclusions and Perspectives

A PBSA for bilingual lexicon extraction is adapted from the standard approach and is based on similarity of context vectors represented by words in a pivot language like English. The PBSA uses two parallel corpora sharing an intermediary language as a pivot language and does not employ any linguistic resources like seed dictionaries. In this paper, we evaluated two different methods for estimating the context vectors under the PBSA in BLE. One estimates them from two parallel corpora on the basis of word association between source words (resp., target words) and pivot words and the other one on the basis of word alignment tools. Though the results were applied to only two language pairs (e.g., Korean-Spanish and Korean-French), the PBSA was quite attractive where public bilingual corpora between two languages are directly unavailable, but public parallel corpora such as English are available. As a result, the method is very promising for resource-poor languages. Furthermore, our methods perform quite well for words with low frequency.

For future works, we plan to extend the pivot-based standard approach to multi-word expressions as well because they play an important role in BLE as mentioned before. Furthermore, evaluation methods against similar words should be considered and more translation equivalents in bilingual dictionary should be added for a larger coverage.

## Figures and Tables

**Table 1:** Comparisons between the standard approach and the PBSA.

Category	Standard	Pivot-based
Corpora for estimating models	Two comparable	Two parallel
Representation of source context vectors	Source words	Pivot words
Representation of target context vectors	Target words	Pivot words
Context vector translation	Required	Not required
Seed dictionary	Required	Not required
Domain adaptation	Relatively easy	Not easy

**Table 2:** The partial output of Anymalign for Korean as a source language and English as a pivot language.

Number	Source word, *s*_*i*_	Pivot word, *e*_*k*_	*Pr*⁡(*e*_*k*_∣*s*_*i*_)	*Pr*⁡(*s*_*i*_∣*e*_*k*_)	Remarks
(1)	*kyeong-chal (police)* ^a^	*police *	0.890421	0.877882	Good
(2)	*kyeong-chal (police) *	*scenario *	1.000000	0.000167	Bad
(3)	*jeong-bu (government) *	*balance *	0.000004	0.000506	Bad

^a^In the second column, Korean words are romanized and are composed of syllables, which are separated by hyphens (-). Words in the parenthesis are their meanings. For example, the meaning of Korean word *kyeong-chal* is *police*.

**Table 3:** An example of a 2 by 2 contingency table for a bilingual word pair, the Korean word *s*_*i*_ = *kyeong-chal* and the English word *e*_*k*_ = *police*.

	*e*_*k*_ = *police*	*e*_*k*_ ≠ *police*	Total
*s*_*i*_ * = kyeong-chal *	*n*_11_ = 80	*n*_12_ = 4,667	*n*_1+_ = 4,747
*s*_*i*_≠* kyeong-chal *	*n*_21_ = 4,510	*n*_22_ = 460,123	*n*_2+_ = 464,633

Total	*n*_+1_ = 4,590	*n*_+2_ = 464,790	*n*_++_ = 469,930

*n*_11_ is the number of times *kyeong-chal* and *police* appears in the parallel corpus.

*n*_12_ is the number of times *kyeong-chal* appears in the Korean text and *police* does not occur in the English text.

*n*_21_ is the number of times *kyeong-chal* does not appear in the Korean text and *police* occurs in the English text.

*n*_22_ is the number of times *kyeong-chal* does not appear in the Korean text and *police* does not occur in the English text.

*n*_1+_ is the number of times *kyeong-chal* appears in the Korean text.

*n*_2+_ is the number of times *kyeong-chal *does not appear in the Korean text.

*n*_+1_ is the number of times *police* appears in the English text.

*n*_+2_ is the number of times *police* does not appear in the English text.

**Table 4:** The average number of words per sentence in the three parallel corpora.

KR-EN		EN-ES		EN-FR	
KR	EN	EN	ES	EN	FR
19.2	31.0	25.4	26.4	27.1	29.7

**Table 5:** The average number of the translations per source word in the evaluation dictionaries.

Evaluation dictionary	HIGH	LOW
KR-ES	5.79	2.26
KR-FR	7.36	3.12
ES-KR	10.31	5.46
FR-KR	10.42	6.32

**Table 6:** A list of Korean translation equivalents for the French word *décision* gotten from the FR-KR evaluation dictionary.

Korean translation equivalent	Frequency^a^	*r*(*t*)
*gyuol-jung* (*decision*)	6,007	0.754
*jae-jung* (*finance*)	880	0.110
*pan-jung* (*judgment*)	414	0.052
*gyeol-ui* (*resolution*)	369	0.046
*gyeol-sim* (*determination*)	173	0.022
*gyeol-dan* (*determination*)	130	0.016
*geol-dan-ryuk* (*the strength of one's mind*)	0	0.000

Total	7,973	1.000

^a^The frequency is counted from the Korean part of the KR-EN parallel corpus.

**Table 7:** Examples of Korean translation candidates for the Spanish word *estrategia* (strategy).

Korean (gloss)	Similarity	Correct answer	Error class
*sa-eop-jeon-ryak (business strategy) *	0.732	False	Compound segmentation
*jeon-ryak (strategy) *	0.725	True	
*su-rip (establishment) *	0.573	False	True negative
*gi-bon-jeon-lyak (basic strategy) *	0.366	False	Compound segmentation
*tu-ja-jeon-ryak (investment strategy) *	0.362	False	Compound segmentation

**Table 8:** Examples of Korean translation candidates for the French word *monde* (world).

Korean (gloss)	Similarity	Correct answer	Error class
*se-gye (world) *	0.929	True	
*se-sang (world) *	0.534	True	
*weol-deu (world) *	0.395	False	Transliterated
*jeon-se-gye (worldwide) *	0.390	False	False positive (de facto true, not in gold standard)
*gak-guk (each country) *	0.307	False	Ditto

**Table 9:** Comparison of the performance in two estimation methods, ANY and CHI.

Frequency	Dictionary	ACC	MRR	PRE	REC	RRC
HIGH	KR-ES	ANY	ANY	ANY	CHI	CHI
	KR-FR	ANY	ANY	ANY	CHI	CHI
	ES-KR	ANY	ANY	ANY	CHI	CHI
	FR-KR	ANY	ANY	ANY	CHI	CHI

LOW	KR-ES	CHI	CHI	CHI	CHI	CHI
	KR-FR	ANY	ANY	ANY	ANY	CHI
	ES-KR	ANY	ANY	ANY	CHI	CHI
	FR-KR	ANY	ANY	ANY	CHI	CHI
